# Temporal and Geographical Variation of Intestinal Ulcers in Grey Seals (*Halichoerus grypus*) and Environmental Contaminants in Baltic Biota during Four Decades

**DOI:** 10.3390/ani11102968

**Published:** 2021-10-15

**Authors:** Bäcklin Britt-Marie, Persson Sara, Faxneld Suzanne, Rigét F. Frank, Roos M. Anna

**Affiliations:** 1 Department of Environmental Research and Monitoring, Swedish Museum of Natural History, P.O. Box 50007, SE 104 05 Stockholm, Sweden; sara.persson@nrm.se (P.S.); suzanne.faxneld@nrm.se (F.S.); anna.roos@nrm.se (R.M.A.); 2 Department of Ecoscience, Danish Centre for Environment and Energy, Arctic Research Centre, Aarhus University, Frederiksborgvej 399, 4000 Roskilde, Denmark; ffr@bios.au.dk

**Keywords:** intestinal lesions, acanthocephalan parasites, herring, guillemot, Baltic Sea, Baltic seal, environmental pollutants

## Abstract

**Simple Summary:**

In the 1970s it was discovered that seal populations in the Baltic Sea had decreased severely due to hunting and high levels of contaminants. Lesions were found in several organs and many of the females became sterile. Since then, most of the organ lesions have decreased and so have the levels of some pollutants. However, ulcers in the large intestines of the grey seals increased in the early 1980s and decreased after the mid-1990s. The aims of this study were to: (1) describe the ulcers and investigate if there is a trend over time that coincides with concentrations of some pollutants in Baltic biota; (2) evaluate the significance of different sea areas in the Baltic, grade of parasite intensity, as well as the sex and age of the seals. The results show that seals with ulcers had, in general, higher parasite intensity. Ulcers were more common in older seals and in the Bothnian Sea. The time trend of ulcers coincides with the trend of certain contaminant levels (BDE-47, PFOS and cadmium). The high prevalence of intestinal ulcers and the high intensity of acanthocephalan parasites appear to be unique to the Baltic population of grey seals.

**Abstract:**

The prevalence of intestinal ulcers and parasites was investigated in 2172 grey seals (*Halichoerus grypus*) collected in the Baltic Sea and 49 grey seals collected outside the Baltic Sea (i.e., the Atlantic). An increase in frequency of ileocaeco-colonic ulcers was observed in the early 1980s, followed by a decrease in the mid-1990s. At the same time, there was an increase followed by a decrease in brominated flame retardants, Perfluorooctanesulfonic acid (PFOS) and cadmium levels in herring (*Clupea harengus*), the most common prey item in Baltic grey seal diet, as well as in another top predator in the Baltic, the common guillemot (*Uria aalge*). The frequency of intestinal ulcers was significantly related to the intensity of acanthocephalan parasites, the age of the seal and the region of the Baltic Sea. Perforation of the intestinal wall was the cause of death in 26 of the investigated Baltic grey seals. In contrast, none of the investigated Atlantic grey seals had intestinal ulcers. They showed a thin colonic wall and very few acanthocephalan parasites. The high prevalence of intestinal ulcers and the high parasite intensity appear to be unique to the Baltic population of grey seals.

## 1. Introduction

The number of grey seals (*Halichoerus grypus*) in the Baltic Sea decreased from 88,000–100,000 specimens at the beginning of the 20th century to approximately 4000 in the late 1970s [1]. Since the 1980s, the number of grey seals has increased to more than 30,000 [2].

At the end of the 1960s, the first traces of polychlorinated biphenyls (PCB) in the Baltic environment were reported [3]. High levels of organochlorines, such as PCBs and dichlorodiphenyltrichloroethane (DDT), were found in ringed seals (*Phoca hispida botnica*) and grey seals from the Baltic [4,5,6]. Apart from hunting, PCBs and DDT were suspected to be factors that contributed to the severe reduction of the Baltic seal populations. High levels of organochlorines were related to findings of aborted seal pups in the southern part of the Baltic [5,7].

The severe reduction in Baltic seal numbers prompted an investigative study at the Swedish Museum of Natural History (SMNH). Seals found dead on land or bycaught in fishing gear have been necropsied at the SMNH since the 1970s. The necropsies revealed a Baltic seal disease complex, comprising certain chronic lesions in the integument, skull bone, intestines, large arteries, kidneys, adrenals and uterus [8,9,10,11,12,13]. An estimated exposure index showed a relation between the prevalence of uterine leiomyomas in the grey seal females and the PCB-level in the Baltic biota [14]. Bergman (1999) reported a significant increase in the prevalence of intestinal ulcers in 1–3-year-old Baltic grey seals from 1987–1996 [11]. Previous bacteriological studies showed no regular findings, which could suggest a common aetiology [15]. There are only sparse reports of colonic ulcers in grey seals outside the Baltic population. An examination in 1988 of 12 grey seals, aged 1–4 years, from the east coast of Scotland, revealed no signs of colonic ulcers and very few ileocaeco-colonic parasites (Bergman, unpublished data). As far as we know, only one case of intestinal ulcers in grey seals outside the Baltic Sea has been reported in the literature [16]. Lakemeyer (2020) reported mild intestinal inflammation by infection of acanthocephalan parasites in North Sea harbour seals and severe infection and colonic ulcers in the Baltic grey seal.

In the mid 1970–1980s, decreasing concentrations of organochlorines were observed in Baltic biota [17,18]. Some fifteen years after the bans on PCBs and DDT, improved reproductive success was noted in the Baltic grey seal, along with decreasing concentrations of PCBs and DDT [19]. Reproductive pathological changes, such as occlusions of the uterine horns, decreased in the 1990s while ileocaeco-colonic ulcers increased. 

The Baltic is known to be polluted by several environmental chemicals. Monitoring of contaminant concentrations in herring (*Clupea harengus*) is conducted yearly at 17 localities in the Baltic as well as in one locality for common guillemot (*Uria aalge*) eggs by the SMNH. Various metals, organochlorines, brominated flame retardants and perfluorinated compounds are analysed. In guillemot eggs, the monitoring of contaminants started in 1969, and for herring the starting period differs depending on locality, but the earliest was in 1978/1980 [20].

Even though concentrations of PCBs and DDTs have decreased in the Baltic [21], other persistent organic pollutants have been discovered, e.g., polybrominated flame retardants (PBDEs) and perfluoroalkylated substances (PFAS) [22,23,24]. Temporal trends of 17 polyfluoroalkyl compounds (PFAS) in grey seals from the Baltic Sea were measured and most of them showed statistically significant increasing concentrations between 1974 and 1997. After 1997 the concentrations of most PFAS decreased or levelled off [24]. In herring from the Baltic, the trend for cadmium was similar to those for PBDE and PFAS, i.e., increasing concentrations from the 1980s, with a peak around 1990s/2000, and decreasing concentrations thereafter [20].

The aim of this study is to describe the morphology of the ulcers and investigate if there is a temporal trend of intestinal ulcer frequency in Baltic grey seals that coincides with temporal trends of environmental pollutants in Baltic biota. Comparisons will be made with concentrations in herring (representing exposure through diet) and guillemot (representing another top predator in the Baltic). In addition, the aim is to evaluate the significance of different geographical regions in the Baltic, intensity of intestinal parasitic infestations, as well as the sex and age of the seals and to make some comparisons with grey seals from the Atlantic. 

## 2. Material and Methods

### 2.1. Specimens

**Figure 1:**
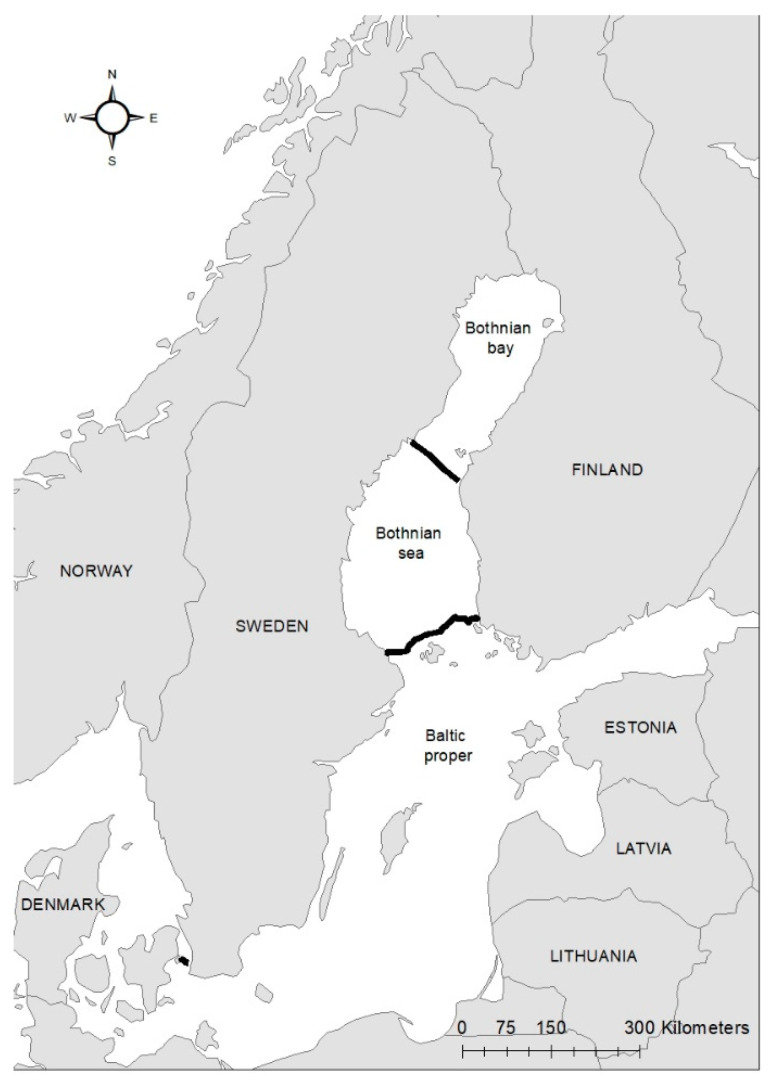
Three sampling areas in the Baltic Sea (Bothnian Bay, Bothnian Sea and Baltic Proper) along the Swedish Baltic coast denoted by black markings. The map was constructed in ArcMap 10.6 using a shapefile downloaded from HELCOM Map (metadata.helcom.fi/geonetwork/, accessed on 11 October 2021) and an ArcGIS online map (Europe-contours pays) from ESRI-France (25 January 2020).

Since the 1970s, fishermen and the general public have been encouraged to send bycaught and stranded seals to SMNH for necropsy and sampling for the Swedish Environmental Specimen Bank. Seals are transported fresh or frozen to the museum. Body weight (kg), length (cm), blubber thickness (mm) and other measurements, such as general health status, reproductive state, etc. and presence of pathological findings, are noted. From 2001, the hunting of grey seals has been permitted in Sweden. Seal hunters are required to send the internal organs, a sample of blubber and the lower jaw with teeth to the SMNH. Since the seals were found dead either on shore or in fishing gear or were legally shot, no ethical permission was required in accordance with Swedish legislation. Hunters and fishermen provide data on when and where the seal was found or shot. In this study, the seals were assigned to three areas along the Swedish Baltic coast; the Bothnian Bay, the Bothnian Sea and the Baltic proper (Figure 1). The grey seals in this study were both males and females, from 0–43 years of age. Age was determined by counting annual growth layers in the cementum of a tooth at the SMNH [25,26]. 

Herring has been sampled and analysed on a yearly basis since 1978/1980 within the national monitoring programme. In this study, data from four stations are used; Bothnian bay (*n* = 622), Bothnian sea (*n* = 590), and two in the Baltic proper (*n* = 608 and 615, respectively).

Here, we have used annual mean value from all localities (*n* = 2435) in order to give a general trend for the whole Baltic, and not potential differences between specific localities.

For more detailed information, see [20,21]. 

In addition, guillemot eggs from Stora Karlsö in the Baltic Proper are also collected and analysed on a yearly basis (1969–2018, *n* = 512). The temporal trends in herring and guillemot are compared with the trends of intestinal ulcers. All chemical analyses have been carried out at Department of Environmental Science, ACES, at Stockholm University. For information about the chemical analyses see [20].

### 2.2. Intestines

**Figure 2:**
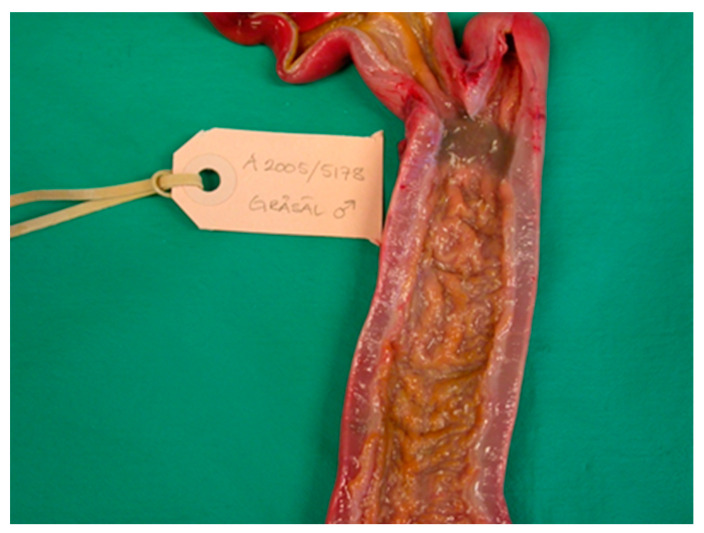
Baltic grey seal ileocaeco-colonic region with a colonic ulcer of moderate degree. The muscular layer of the intestinal wall has thickened.

**Figure 3:**
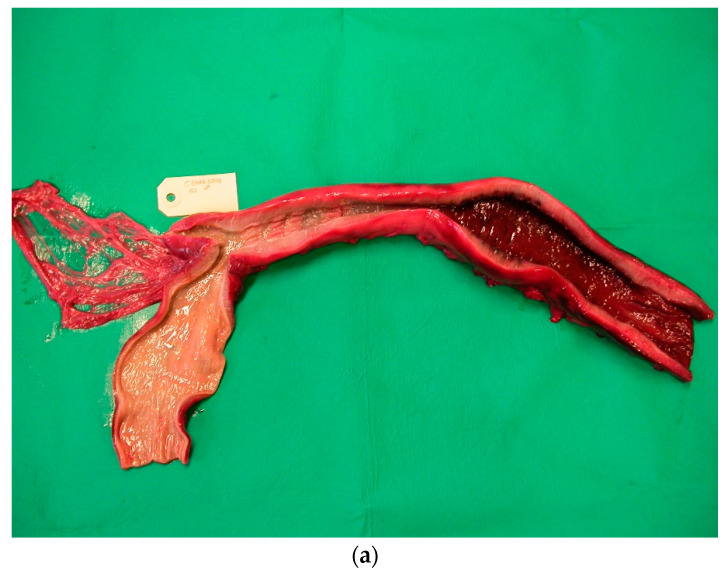

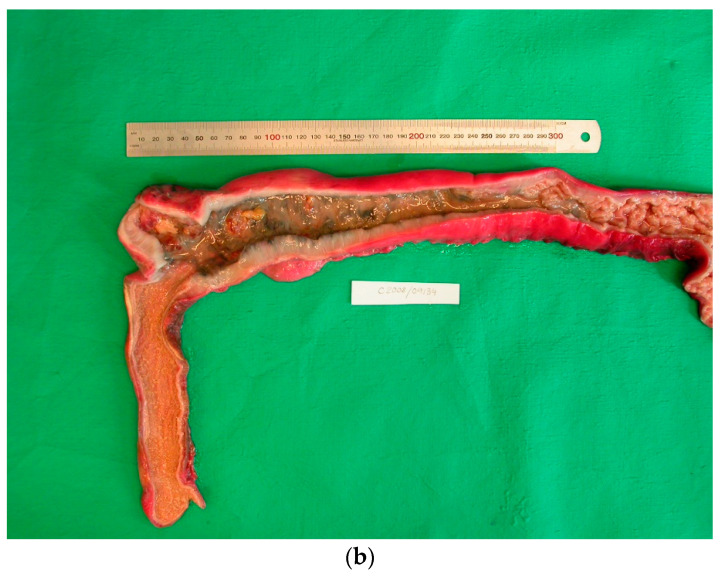
(**a**,**b**). Baltic grey seals with severe colonic (**a**) and caeco-colonic (**b**) ulcers. The muscular layer of the intestinal wall has thickened.

**Figure 4:**
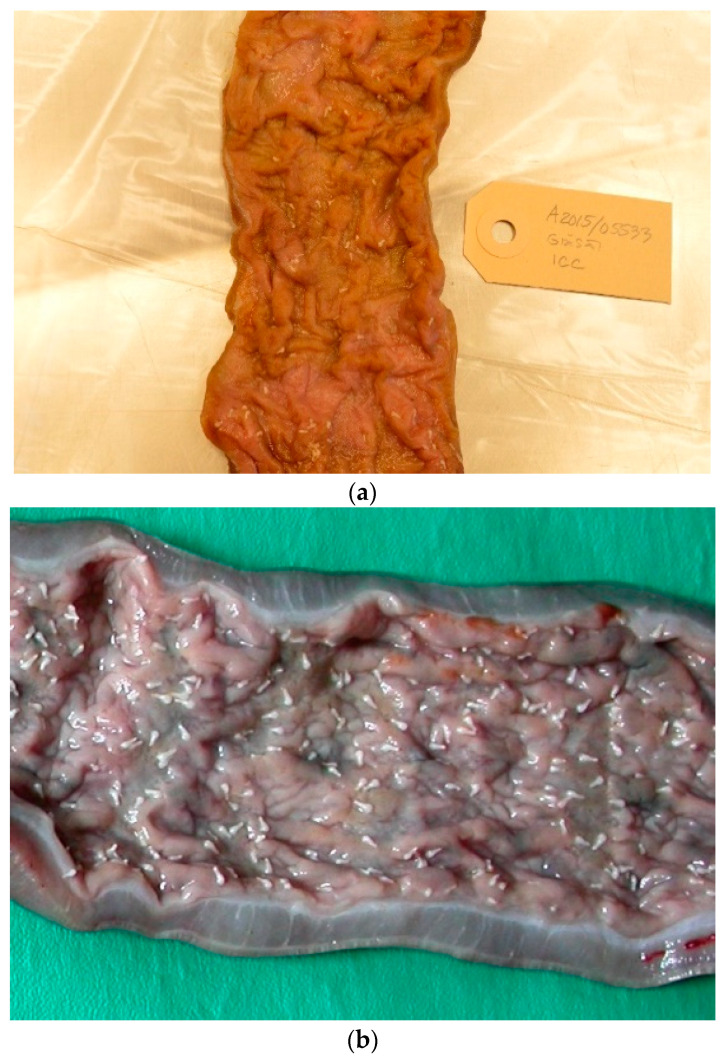

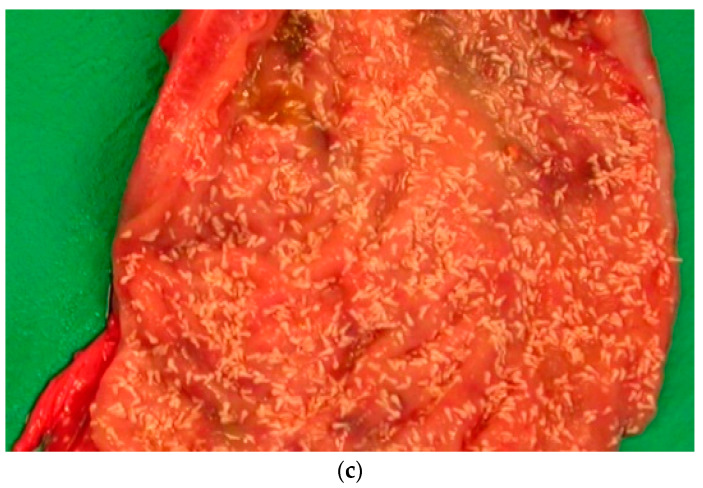
(**a**–**c**). Intensity of colonic acanthocephalan parasites, estimated as slight (**a**) moderate (**b**) and high (**c**).

From 1977 to 2016, the intestines of 2172 grey seals from the Baltic Sea (Figure 1) were macroscopically investigated, intestinal ulcers were noted and measured and the prevalence of the acanthocephalan parasite *Corynosoma* spp. was estimated. After 2002, the thickness of the colonic muscular layer was also measured. The valuation of intestinal ulcers and parasites was made as in Bergman [11] and Bäcklin et al. [27]. Erosions, a delimited minor decrease in the mucosal thickness, and ulcers, a delimited deeper decrease in the mucosa were evaluated on a scale of 0–3: <4 mm erosions (0); 4–10 mm erosions or ulcers (1); > 10 mm erosions or ulcers (2); and >10 mm ulcers also affecting the muscular layer (3). Only the prevalence of intestinal lesions of moderate (2, Figure 2) and severe (3, Figure 3a,b) degree, i.e., lesions exceeding 10 mm in diameter were considered to be pathological [11] and are here referred to as intestinal ulcers. The number of colonic acanthocephalan parasites was evaluated as none (0), slight (1), moderate (2) or high (3) intensity (Figure 4a–c). 

Intestinal samples from a subset of 10 Baltic grey seals were studied in light microscope. Tissue samples were fixed in 4% neural buffered formaldehyde, embedded in paraffin wax and sectioned at 5 µm. Sections were stained with haematoxylin and eosin (H&E) and Masson’s trichrome.

In addition, intestines from 49 grey seals from the north Atlantic, sent frozen to the SMNH, were investigated. From Canada, specifically, the Department of Fisheries and Oceans in Quebec, we received samples from 10 females (4–24-years-old) and 13 males (0–23-years-old). From the Institute of Marine Research in Tromsø, Norway we received 4 females 158–218 cm and 6 males 151–225 cm in body length. From BioPol Marine Biotechnology Company in Iceland, we received 16 specimens, 111–173 cm in body length. The age of the seals were received from Canada and the body length were received from Norway and Iceland. All of them were shot during the hunting season in 2006–2008. 

### 2.3. Contaminant Analyses

Thanks to the extensive national monitoring program of contaminants in biota in Sweden, data in guillemot eggs and herring are available for many contaminants. The guillemot is a top predator in the marine environment in Baltic Proper and therefore it can be assumed that trends in this species also reflect trends in other top predators in the Baltic Sea. In addition, herring, which is collected at several localities along the Swedish coastline, can be used to verify if trends seen in guillemot eggs are also found in herring. In addition, herring is also an important part of the diet of grey seals. In this study, trends of analysed contaminants that followed the same trend as intestinal ulcers are presented. Additionally, PCB and DDT levels are presented since the levels were high when the Baltic seal disease complex were discovered. We used data on ∑PCB, the brominated flame retardant BDE-47, and perfluorooctane sulfonic acid (PFOS) in guillemot egg and herring collected annually in the Baltic Sea since 1969 and 1980, respectively. Moreover, cadmium concentrations in herring liver were also used. PCB and BDE-47 was analysed in fish muscle and PFOS in fish liver. 

### 2.4. Statistics

Statistical analyses were performed using the software R Development Core Team 2017 Version R 3.4.0 [28]. The yearlings (i.e., animals under one year old), 591 of the 2172 investigated Baltic grey seals, were not included in the trend analyses since many of them were too young to have started feeding on fish. Although contaminants could be transferred through lactation, the acanthocephalan parasite intensity will differ between the nursing pups and weaners from the older ones since the parasites are transferred from fish.

The occurrence of intestinal ulcers did not depend on sex (χ^2^ test, *p* = 0.48) or cause of death (bycaught or hunted) (χ^2^ test, *p* = 0.23) and these were therefore not considered further. 

Generalized linear models (GLM) assuming a binomial error structure were applied to test the influence of age, year, area and acanthocephalan parasite infection grade on the occurrence of intestinal ulcers in bycaught and hunted seals. Parasite intensity was classified as: 0 and 1: no and slight intensity; 2: moderate; and 3: high intensity. Besides these explanatory variables, quadratic terms of both year and age were included in the model to test whether quadratic relationships with the occurrence of intestinal ulcers were significant. Only data from the Bothnian Bay, Bothnian Sea and Baltic Proper were included in these analyses, and not the seals from the Atlantic. First-order interactions between the variables age, area and acanthocephalan parasite infection grade and year were included as this study covers a period of four decades and changes with time may be expected, as well as interaction between area and acanthocephalan parasites. Based on the first run of this model, non-significant (*p* > 0.05) variables were removed and the model re-un. The reduced final model that included the explanatory variables area, age, year, year^2^, parasite infection grade and the interaction between year and area, was tested against the original model using the chi-square test. Based on the final GLM model, a prediction of the occurrence of ulcers in intestines with no, slight, moderate or high intensity of parasites was made. See Supplementary Information Table S1.

A statistically robust method was applied to the contaminant time-series data. Briefly, annual median concentrations were used as the index values. The method tests for the presence of a log-linear trend and/or non-linear trend by separating the total variance over time into a log-linear component and a non-linear component [29]. The log-linear trend was tested by log-linear regression. A 3-year running smoother was applied to describe the non-linear trend component and tested by means of an analysis of variance (ANOVA), α < 0.05 was applied.

Student’s two sided t-test was used to test the mean colonic muscular layer thickness between intestines with no or moderate ulcers and those with severe ulcers.

## 3. Results

### 3.1. Morphology of the Intestinal Lesions 

**Figure 5:**
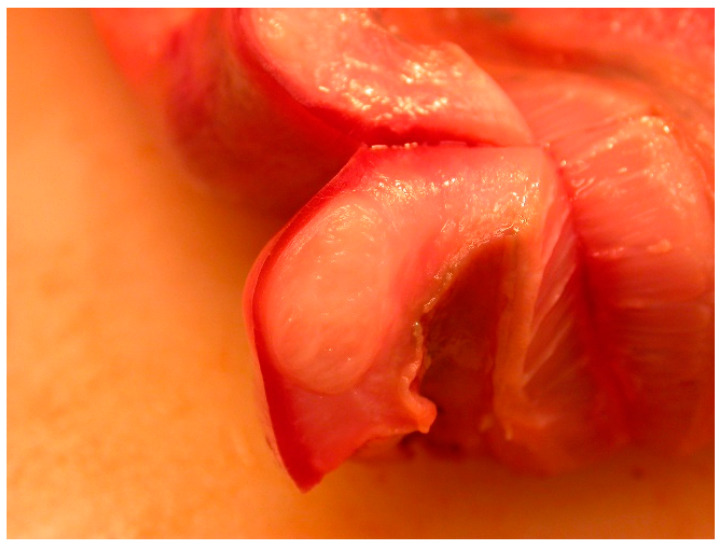
Colonic leiomyoma with an oval white, firm texture in the adjacent thick muscular layer in a Baltic grey seal.

**Figure 6:**
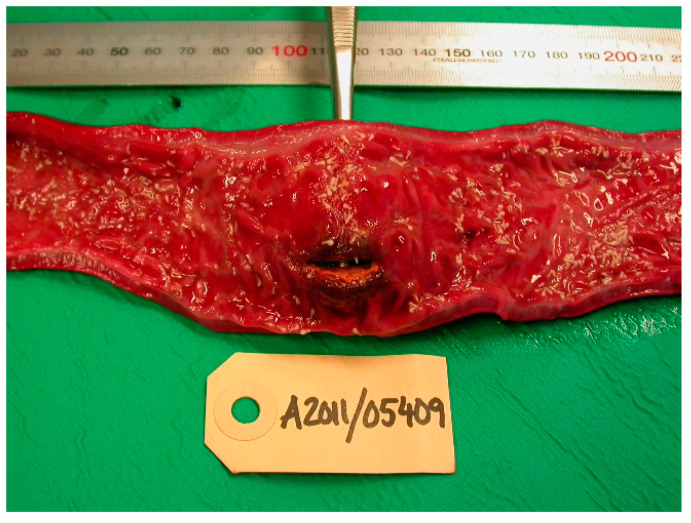
Perforation of the colonic wall in a Baltic grey seal.

The intestinal lesions were mainly located in the ileocaeco-colonic region, predominantly in the anterior part of the colon. The intestinal lesions were solitary or multiple, often confluent areas with erosion of the mucosa. The erosions were mainly localized along the tips of the folds of the colon. The size of the intestinal ulcers could vary from a few millimetres in diameter (degree 1) to extended ulcers occupying large parts of the ileum and colon (degree 2–3). Old and recent haemorrhages were common in the ulcers and, in severe cases, also in the muscular layer of the affected area. The muscular layer of the diseased part of the intestine was often thickened. In the muscular layer, leiomyomas measuring 5–20 mm were found in a few cases (Figure 5). Perforation of the intestinal wall was the cause of death in 26 of the investigated Baltic grey seals (Figure 6). One seal was a yearling, and the other seals were 1–37 years old (17 males, 8 females). 

Histological sections of colonic ulcers revealed a necrotic or shed mucosa. The villi of the colonic mucosa near the ulcers often displayed a thickened or fused appearance. The parasites were attached to the mucosa by proboscial hooks and desquamation of epithelial cells was prominent in the attachment site. In general, the lamina propria was often thickened and the inflammatory response was mild and mainly comprised of lymphocytic cells and eosinophils. The muscularis mucosa and the submucosa were often oedematous and thickened. In the colonic wall, it was mainly the inner muscular layer that was thickened. 

**Figure 7:**
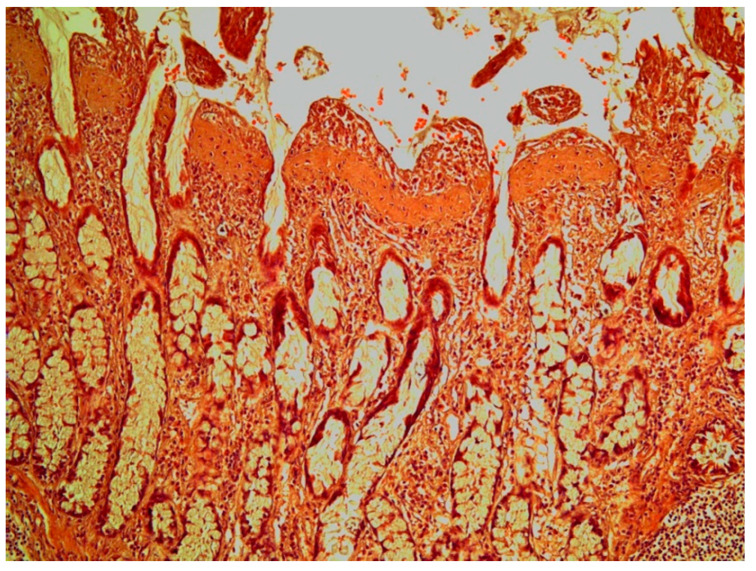
Colonic mucosa in a grey seal showing thickened villi with a subepithelial collagen band. Haematoxylin & Eosin × 10.

In the histological sections of two specimens with severe colonic ulcers, subepithelial, thickened villi showed a band of connective tissue (Figure 7) that stained positive with Masson´s trichrome for collagen. The subepithelial collagen band was evident in both the ileum and the colon. 

In the studied Baltic grey seals, a high number of acanthocephalan parasites (*Corynosoma* spp.) were often present in the ileocecal-colonic region, and to a greater or lesser extent, in and around the affected areas. Even when flushing the intestinal region of faeces with water, the parasites were still tightly adhered to the mucosa. 

Although yearlings were not included in the statistical calculations, it should be noted that 16% (93/591) of the studied yearlings from 1977–2016 had moderate to severe colonic ulcers. Among the yearlings with moderate to severe colonic ulcers, 79% had moderate to severe infection with *Corynosoma* spp. Yearlings without colonic ulcers showed a lower level, 45%, of moderate to severe infection with *Corynosoma* spp. 

**Figure 8:**
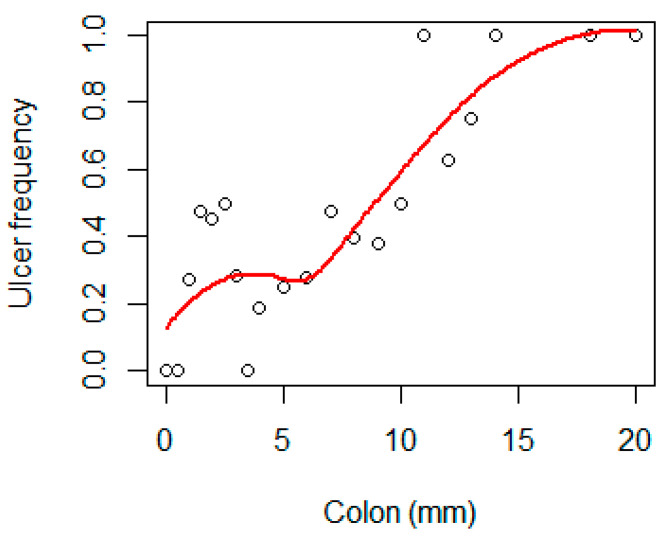
The frequency of ulcer versus colonic muscular layer thickness. The red line represents a loess smoother line.

The mean colonic muscular layer was significantly thicker in seals with moderate to severe ulcers compared to seals with or without minor erosions (t-test, *p* < 0.001, Figure 8). Additionally, the mean thickness of the muscular layer was significantly correlated to the degree of parasitic infection (r = 0.83, *p* = 0.004).

The following variables were included in the final GLM analyses of the occurrence of intestinal ulcers: area, age, year, the quadratic term of year, the occurrence of parasites (i.e., no or mild parasitic infection vs. moderate to severe infection) and interaction between year and area. The reduced final model did not result in a significant loss of the model performance (*p* = 0.20). See Supplementary Information Table S2.

### 3.2. Frequency of Ulcer in Relation to Age

**Figure 9:**
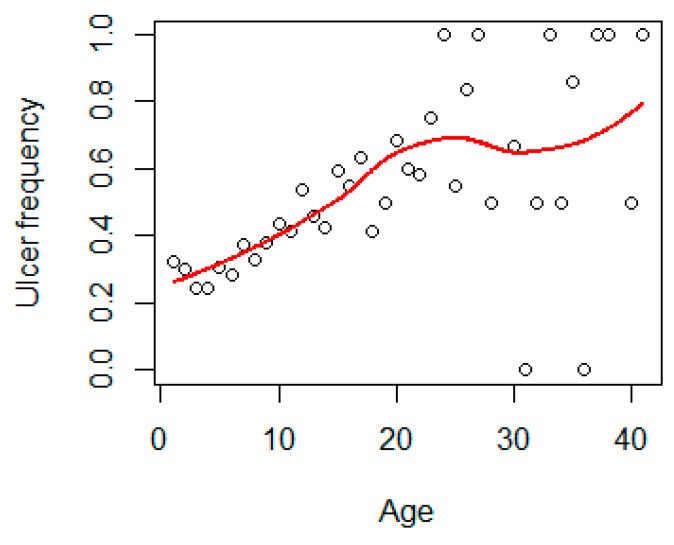
Mean frequency of ulcers versus age in Baltic grey seals. The red line is a loess smoother line.

In general, the frequency of ulcers increased with age up to approximately 20 years of age (Figure 9). 

### 3.3. Temporal and Spatial Trends of Parasites and Ulcers

**Figure 10:**
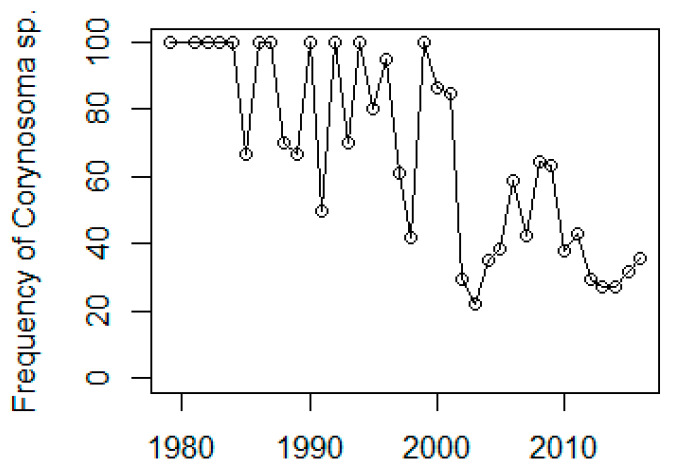
Frequency of moderate to high acanthocephalan parasite intensity versus year.

The temporal trend of intestinal ulcers was similar for the three areas with a decreasing frequency of ulcers after the mid-1990s. The frequency of moderate and severe infections of acanthocephalan parasites (degree 2 and 3) was high throughout the 1980s and 1990s, but then decreased around the year 2000 (Figure 10). 

**Figure 11:**
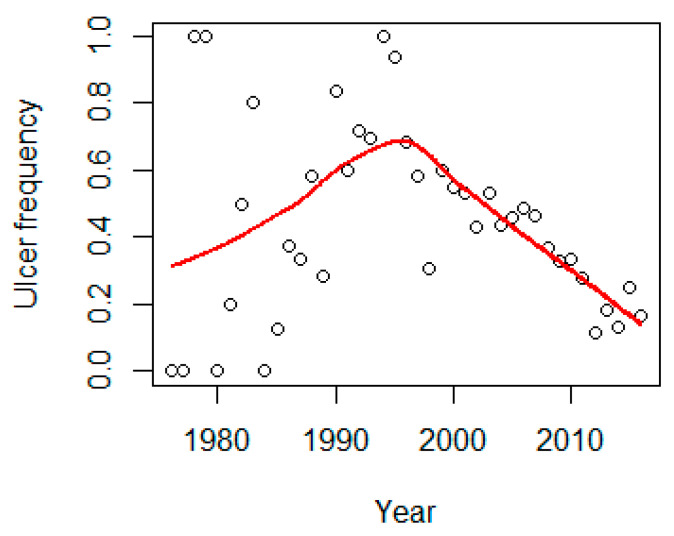
The frequency of ulcers increased up to the mid-1990s and then a decrease is seen. The red line is a loess smoother line.

The year and the quadratic term of year showed a highly significant (*p* < 0.001) influence on the frequency of ulcers. The mean annual occurrence of intestinal ulcers as predicted by the GLM model showed an increase up to the mid/late 1990s followed by a decrease until 2016 (Figure 11). In 2016, the predicted frequency was 0.12, the lowest in the whole time-series. Similarly, age significantly (*p* < 0.001) influenced the frequency of ulcers (Figure 9). 

**Figure 12:**
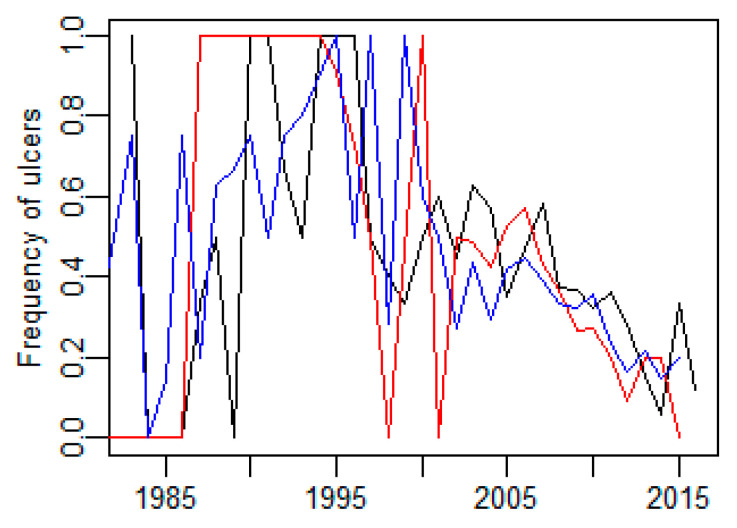
Frequency of ulcers in grey seals from Baltic Proper (blue), Bothnian Sea (black) and Bothnian Bay (red).

The area and interaction between year and area significantly influenced the frequency of ulcers (*p* < 0.001 and *p* = 0.02, respectively). Generally, the frequency of ulcers was lowest in the Baltic Proper (23%) and highest in the Bothnian Sea (43%) and in-between in the Bothnian Bay (38%). The significant interaction was caused by the relatively low number of samples, especially in some of the early years giving high year-to-year variation in the three areas. Since the mid-1990s, the temporal trend of the frequencies in the three areas has been similar and has decreased from around 50% to below 20% in 2016 (Figure 12).

The frequency of ulcers was significantly influenced by the amount of acanthocephalan parasites (*p* < 0.001). The mean frequency of ulcers in the 3-degree scale of parasite intensity predicted from the statistical model, was 25% with no or slight, 40% with moderate and 58% with high parasite intensity. See Supplementary Information Table S3.

### 3.4. Temporal Trends of Contaminants 

∑PCB showed downward trends in both guillemot egg and herring over the whole time series, with annual decreases of 9.1% and 5.5%, respectively. A similar pattern was also seen for DDE in the two species (see Supplementary Information Figures S1 and S2). 

**Figure 13:**
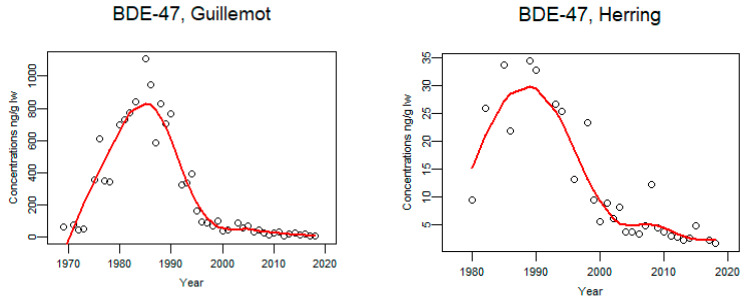
Time trends of BDE-47 (ng/g lw) in guillemot egg (*n* = 306) from the Baltic proper 1969–2018 and herring muscle from the Baltic 1980–2018 (median annual values from 4 localities, *n* = 217). The circles represent annual median values and the red line shows a smoother.

Concentrations of BDE-47 increased in guillemot eggs from 1969 until the mid-1980s and since mid-1990s the concentrations have been decreasing (Figure 13). In herring, concentrations of BDE-47 increased from 1980 to mid-1990 and thereafter concentrations are decreasing (Figure 13); thus, we see a similar pattern as in guillemot eggs.

**Figure 14:**
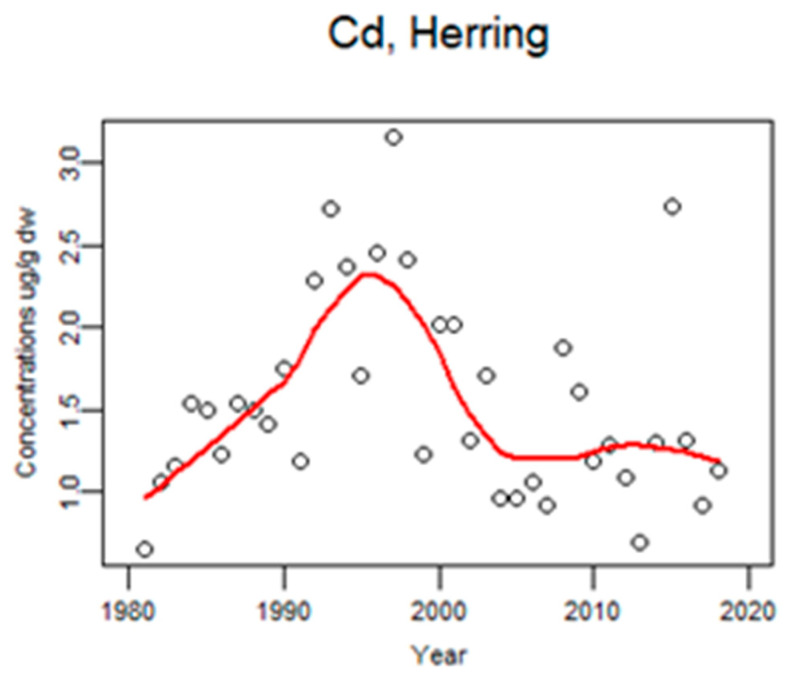
Time trends of Cd (ug/g dw) in herring liver from the Baltic 1981–2018 (median annual values from 4 localities, *n* = 1109). The circles represent annual median values and the red line shows a smoother.

Additionally, cadmium showed increasing levels in herring up to the late 1990s followed by a decrease (Figure 14). Concentrations of Cd in guillemot eggs were very low, close to the limit of detection (LOD) and, thus, a time trend is not shown.

**Figure 15:**
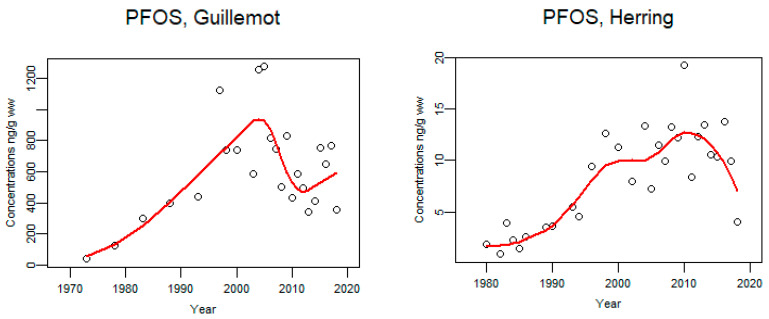
Time trends of PFOS (ng/g lw) in guillemot egg (*n* = 24) from, Baltic proper 1973–2018 and herring liver from the Baltic 1980–2018 (median annual values from 4 localities, (*n* = 123). The circles represent annual median values and the red line shows a smoother.

PFOS in guillemot eggs showed an increase from 1973 until approximately 2002 and thereafter concentrations have decreased but seem to have levelled out after 2010 (Figure 15). In herring, concentrations of PFOS increased from 1980 until around 2000 and after that concentrations have stayed fairly stable for many years (Figure 15).

### 3.5. Observations of Atlantic Grey Seal Intestines 

**Figure 16:**
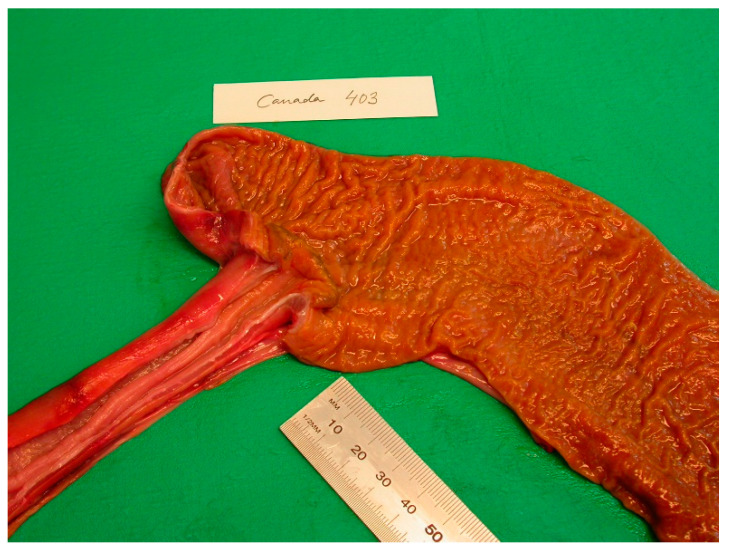
The ileocaeco-colonic region of an Atlantic grey seal.

No mucosal erosions or ulcers were observed in any of the investigated grey seal intestines from Canada, Norway and Iceland. Furthermore, the number of acanthocephalan parasites in the colonic region of these seals was only 0–7 individual parasites and the colonic muscular layer was only 1–2 mm thick. No increase in parasite intensity or thickness of the colonic muscular layer with age (or size) of the seals were seen in the Atlantic grey seals (Figure 16).

## 4. Discussion

The series of health monitoring data of more than 2000 grey seals during four decades, in combination with contaminant monitoring data, revealed temporal trends of pathological changes in relation to contaminant data. 

The frequency and severity of intestinal ulcers are unique for grey seals in the Baltic. In consistence with a study by Lakemeyer et al. (2020) on Baltic grey seal intestines [30], the frequency of intestinal ulcers and parasite intensity increased with the seals’ age. In contrast to our study, there was a difference between the sexes, with a higher intensity in males. The parasite intensity in the colonic part of the intestine could be very high. In an earlier study of 48 Baltic grey seals (also included in the present study), a mean intensity of 1000 *Corynosoma* (mainly *C. semerme*) in the ileocaeco-colonic region was recorded and the highest number of parasites was 5400 in a 23-year-old grey seal male [31]. In an Irish study the mean abundance was only 416 and the range 80–846 of *C. strumosum* in the total length of the intestines in 26 Atlantic grey seals [32]. 

At least 15 fish species in the Baltic Sea have been reported to have *Corynosoma* infection [33]. The Baltic grey seal feeds to 80% on herring but also sprat (*Sprattus sprattus*), and common whitefish (*Coregonus lavaretus*) are usual dietary items as well as cod (*Gadus morhua*) when available [34]. The most common species of *Corynosoma* in the caecum and colonic region in the Baltic grey seal, *C*. *semerme*, have mainly been found in European smelt (*Osmerus eperlanus*), herring, cod and flounder (*Platichtys flesus*) [35]. Whether or not the parasite intensity of *Corynosoma* spp in herring has changed over time has, to our knowledge, not been studied.

The prey items differ some between the north (Bothnian Bay and Bothnian Sea) and south (Baltic proper) Baltic Sea although the principal prey in both areas are herring. In the northern part, salmon (*Salmo salar*), trout (*Salmo trutta*) and sculpins (*Cottoidea* spp) have also been identified in stomach content, while cod and flatfish were only identified in stomachs from the southern part of the Baltic [34].

In general, intestinal lesions are usually initiated by the destruction of the epithelial cell barrier by bacteria, virus, toxins or parasites. Two types of inflammatory bowel disease described in humans are ulcerative colitis and Crohn´s syndrome. The cause of these diseases is unknown. Evidence thus far in humans suggests an abnormal immune response to microorganisms in genetically susceptible individuals [36]. The results from animal models and human studies indicate a role for innate lymphoid cells at intestinal mucosal sites in protecting and inducing inflammation against bacteria, parasites and viruses [37,38]. In the present study, the observed histological responses to the intestinal parasites mainly comprised a thin or absent mucosa and mild infiltration of lymphocytic cells and eosinophils close to the attachment sites of the parasites. Other studies have found only a slight intestinal inflammatory response to *Corynosoma* in seals and the attachment pattern of *Corynosoma* observed in South American fur seals significantly reduced the thickness of the intestinal mucosa [39,40]. Minor intestinal lesions (erosions less than 10 mm in diameter) in Baltic grey seals are supposedly unavoidable due to the attachment of the parasites, although larger ulcerations are likely to be pathological. Lesions in the mucous membrane caused by parasites destroying the epithelial barrier of the ileocaeco-colonic region are interpreted as being the primary event of the ulcerous processes in Baltic grey seals. In two cases a collagen deposition was found subepithelially in the ileo-colonic mucosa. In humans, a microscopic diagnosis of collagenous colitis was first described in 1976 when a thick subepithelial collagenous deposit in the colorectal mucosa was identified in a rectal biopsy from a patient with chronic diarrhoea [41]. The cause of collagenous colitis in humans is not known but it has been seen in patients with a history of autoimmune or coeliac disease and certain medications have been suggested as possible triggers [42]. 

The age-related parasite intensity in the present study was in accordance with other studies on Baltic seals [30,43,44]. Observations of an increase in the number of *C. strumosum* with age have also been seen in harbour seals from the southern parts of the Baltic [44] and in Saimaa ringed seals (*Pusa hispida saimensis*) [45]. In a study of Atlantic grey seals, *C. strumosum* was the only acanthocephalan species found in the intestine and they were localised to the small intestine [32]. In the present study, grey seal intestines from Canada, Norway and Iceland showed only a few individuals of *Corynosoma* spp. in the ileocaeco-colonic region, irrespective of age. These results and previous studies show a difference in the intensity of infection as well as in the presence of *C. semerme* in Baltic compared to Atlantic grey seals. 

The observed thickening of the colonic muscular layer in the Baltic grey seal may be a response to a chronic exposure to high parasite intensity and the ulcerous process in combination with suppression of the immune system and environmental contaminants. In chronic inflammatory diseases, the inflammatory cascade and healing involves cytokines released from epithelial cells that activate inflammatory cells and initiate fibrosis and smooth muscle cell hyperplasia [46].

The increase and decrease in ulcer prevalence over time in all three areas of the Baltic Sea and higher frequency in the Bothnian Sea compared to the other two areas indicate influence of environmental factors and/or changes in the ecosystem. The diet differ somewhat between the northern and southern area of the Baltic but the main prey in the whole area is herring [34]. Contaminants have been extensively monitored and levels of organochlorines, such as PCBs and DDTs, have decreased significantly during the last three to four decades (Figures S1 and S2) [21].

Immunological reactions to parasites have most probably co-evolved with the host in order to ensure that both of them survive. In chronic parasitic infections, parasites have the ability to dampen the immune response for co-existence with the host [46]. Thus, the severity and the extent of the affected areas of the colonic mucosa and the high intensity of *Corynosoma* spp. in the specimens could be signs of a defective immunological process.

The contaminants presented in this study all have immunosuppressive effects [47,48,49,50,51,52,53]. Effects in rats and rodents after exposure to PentaBDE (including BDE-47 and BDE-99) were changes in hepatic and thyroid size and histology, and altered serum thyroxin levels [54]. Exposure of environmentally relevant PBDEs levels to harbour seal (*Phoca vitulina*) granulocytes caused oxidative stress by depleting the level of thiols and/or increasing reactive oxygen species production and corresponding changes in phagocytic activity and efficiency [55]. In addition, exposure studies with PBDEs in mice have revealed a suppressed immune system when they were infected [47]. The results of an in vitro study with harbour seal leucocytes and PBDEs suggest that the immune response of harbour seals exposed to PBDEs could fail to respond appropriately when invaded by microorganisms [55]. Furthermore, there is a connection between PFAS exposure and health problems, including suppressed immune function, lower vaccine effectiveness, hypersensitivity and greater risk of autoimmune diseases [48,56,57,58]. In addition, PFOS and perfluorooctanoic acid (PFOA) are potential developmental toxicants and are suspected endocrine disruptors [59]. In a study on bottlenose dolphins, the number of eosinophils and the concentration of some acute phase proteins in the blood significantly decreased with increasing concentrations of PFOS [60]. Eosinophils are central in the immune response to helminth infection. They generally regulate multiple inflammatory mechanisms, including a wide spectrum of cytokines that can influence T cell differentiation, tissue repair or amplify the inflammatory response with the risk of enhancing tissue damage [61]. Interestingly, PFOS treatment in mice led to a failure to clear a bacterial infection, showing increased bacterial counts along with increased inflammatory cytokines, reduced mucin production and dysbiosis [62]. The effect of environmental pollutants on the immune system of marine mammals has been thoroughly reviewed [63] and reveals effects of PCB, as well as cadmium and mercury. Similarly, the immune system suffers from cadmium induced impairment at several levels [49,50,51].

While the trend of PCB in Baltic biota does not seem to coincide with the trend for intestinal ulcers, the possible influence of PCB should not be overlooked as the overall level of contamination is high in the Baltic. In vivo and in vitro studies have shown that PCB interfere with intestinal epithelial cells and endothelial cells and disrupt the barrier integrity of the intestines and blood vessels [64,65,66,67]. In this study, the trends of BDE-47 and PFOS in both guillemot eggs and herring, as well as cadmium in herring, coincide with the trend for intestinal ulcer in Baltic grey seals. Thus, BDE-47, PFOS and cadmium could all be drivers of the risk for intestinal ulcers in seals in the Baltic Sea. In summary, together with infection by a specific parasite and possibly an altered intestinal biome, several contaminants have the ability to compromise the immune response in the gut and may contribute to the problem of colonic ulcers in Baltic grey seals. While the frequency has decreased over time, the situation seems to persist, with approximately 10–20% of the investigated seals affected each year. Further studies of environmental factors, such as levels of contaminants and mixtures of contaminants in the seals, are required. 

## 5. Conclusions

The frequency of Baltic grey seals with intestinal ulcers increased up to the mid-1990s and then decreased until 2016. Since around 2000, there has been a decrease in Baltic grey seals with moderate and high intensity of intestinal acanthocephalan parasites. The frequency of ulcers increased with the parasite intensity. Even so, 25% of seals with a mild amount of parasites had ulcers. The frequency of intestinal ulcers increased with the age of the seal, although 16% of the yearlings were also affected. Since there is a clear difference in the frequency of ulcers over time, we suspect that an underlying cause for the ulcers could be an environmental factor, for example environmental pollutants or unknown pathogens. The trend seen in frequency of ulcers over time is very similar to the trend found for BDE-47 and PFOS in herring and guillemot eggs and cadmium in herring from the Baltic. However, if any of these compounds, alone or in combination, is the underlying cause of the severity of the ulcers remains to be evaluated. Finally, no grey seal intestines from the Atlantic had ulcers, a thick muscular layer or moderate to severe parasitic infections. The high intensity of *Corynosoma* spp. in the ileocaeco-colonic region, as well as the accompanying ulcerations, appear to be unique to the Baltic grey seals. 

## Data Availability

The data presented in this study are openly available for sealpathology in https://sharkweb.smhi.se/hamta-data/ (accessed on 11 October 2021) and for herring and guillemot in https://www.sgu.se/produkter/geologiska-data/nationella-datavardskap/datavardskap-for-miljogifter/rapporterad-data-till-datavardskap-for-miljogifter/ (accessed on 11 October 2021).

## Supplementary Materials

The following are available online at https://www.mdpi.com/article/10.3390/ani11102968/s1, Figure S1: Time trends for ∑PCB, Figure S2: Time trends for DDE, Table S1: Results of final GLM, Table S2: Results of ANOVA, Table S3: Estimated frequences parasite intensity and intestinal ulcers.
