# Supplementary material for: Temporal and Geographical Variation of Intestinal Ulcers in Grey Seals (Halichoerus grypus) and Environmental Contaminants in Baltic Biota during Four Decades
Source: Animals (Basel). 2021 Oct 15;11(10):2968. doi: 10.3390/ani11102968 (PMC8532654; doi:10.3390/ani11102968)
Supplement: Supplementary file 1 [file animals-11-02968-s001.zip › animals-1383208-supplementary.pdf]

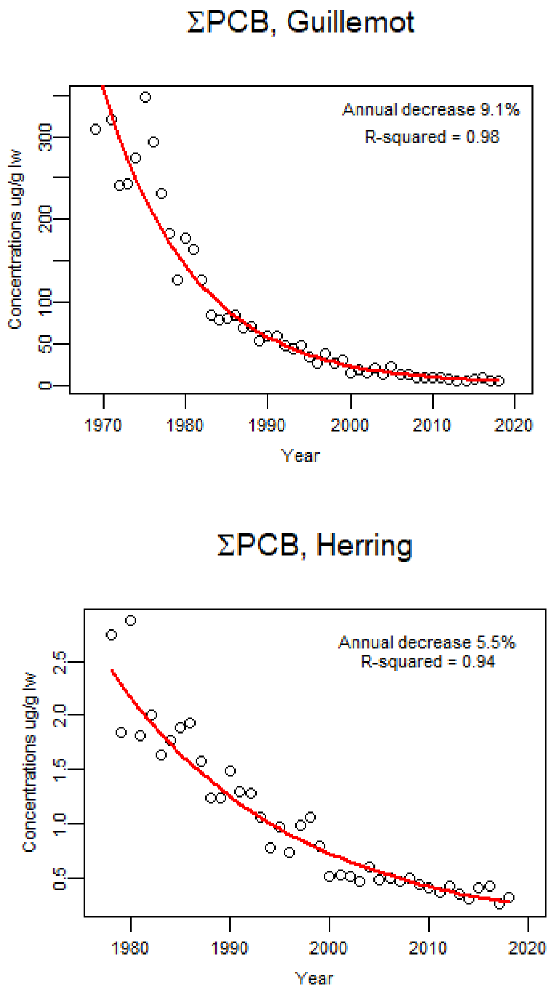

Figure S1. Time trends for ∑PCB (µg/g lw) in guillemot egg from Baltic proper 1969-2018 (n=513) and herring muscle from the Baltic 1978-2018 (median annual values from 4 localities, n=2435). Circles represent annual median values and the red line represents a log linear regression line.

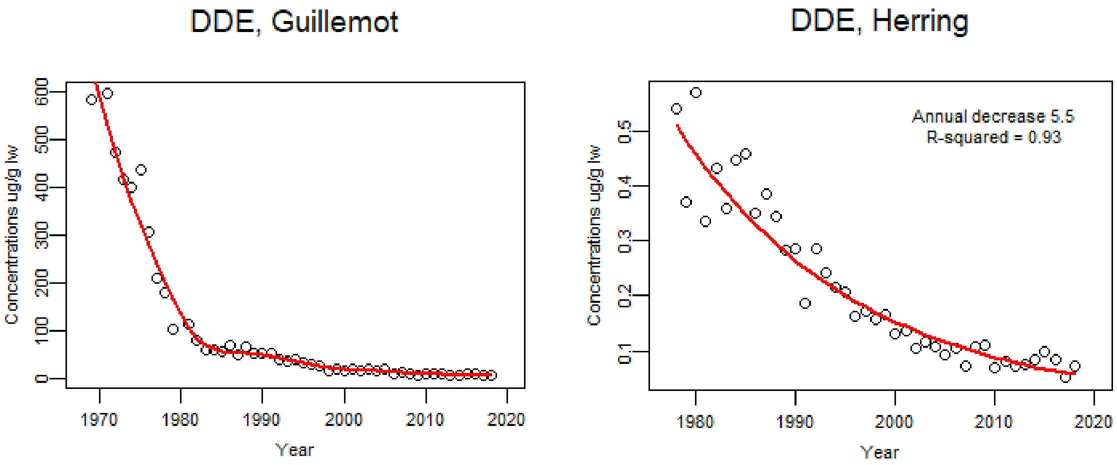

Figure S2. Time trends for DDE (µg/g lw) in guillemot egg from Baltic proper 1969-2018 (n=513) and herring muscle from the Baltic 1978-2018 (median annual values from 4 localities, n=2435). Circles represent annual median values and the red line represents a log linear regression line.

Table S1. Results of final GLM with a binomial error structure.

Variable	Df	Deviance	Residual Deviance	P(>Chi)
Null variable			1866.2	
Area	2	50.08	1816.2	<0.001
Age	1	46.90	1769.3	<0.001
Year	1	54.02	1715.2	<0.001
$Year^2$	1	30.87	1684.4	<0.001
Parasite intensity		1	35.30	1649.1 <0.001
Area*Year	2	7.69	1641.4	0.021

Table S2. Results of ANOVA for comparison of full and reduced final model.

Variable	Residual Df	Residual	Deviance	P(>Chi)
Full model	1426	1632.8		
Final model	1430	1641.4	-8.62	0.074

Table S3. Estimated frequencies based on predictions of the final statistical model.

Parasite intensity	Frequency of ulcers
No or slight	25%
Moderate	40%
High	58%
